# Atorvastatin Protects Against Deleterious Carfilzomib-Induced Transcriptional Changes in Human Induced Pluripotent Stem Cell-Derived Cardiomyocytes

**DOI:** 10.3390/ijms27031358

**Published:** 2026-01-29

**Authors:** Marwa Tantawy, Danxin Wang, Mohammed Gbadamosi, Fahong Yu, Yanping Zhang, Mohammed E. Alomar, Kenneth H. Shain, Rachid C. Baz, Katelyn A. Bruno, Yan Gong

**Affiliations:** 1 Department of Pharmacotherapy and Translational Research and Center for Pharmacogenomics and Precision Medicine, College of Pharmacy, University of Florida, Gainesville, FL 32610, USAdanxin.wang@cop.ufl.edu (D.W.); mgbadamosi@cop.ufl.edu (M.G.); 2 Cardio-Oncology Working Group, University of Florida Health Cancer Institute, Gainesville, FL 32610, USA; katelyn.bruno@medicine.ufl.edu; 3 Bioinformatics at Interdisciplinary Center for Biotechnology Research, University of Florida, Gainesville, FL 32610, USA; fyu@ufl.edu; 4 Gene Expression & Genotyping at Interdisciplinary Center for Biotechnology Research, University of Florida, Gainesville, FL 32610, USA; yanp@ufl.edu; 5 Cardio-Oncology Program, H. Lee Moffitt Cancer Center & Research Institute, Tampa, FL 33216, USA; mohammed.alomar@moffitt.org; 6 Division of Cardiovascular Sciences, Morsani College of Medicine, University of South Florida, Tampa, FL 33620, USA; 7 Department of Malignant Hematology, H. Lee Moffitt Cancer Center & Research Institute, Tampa, FL 33216, USA; ken.shain@moffitt.org (K.H.S.); rachid.baz@moffitt.org (R.C.B.); 8 Division of Cardiovascular Medicine, Department of Medicine, University of Florida, Gainesville, FL 32610, USA

**Keywords:** carfilzomib, cardiotoxicity, induced pluripotent stem cells, atorvastatin

## Abstract

The mechanisms underlying carfilzomib (CFZ)-induced cardiotoxicity remain incompletely elucidated. In this study, we used human induced pluripotent stem cell-derived cardiomyocytes (hiPSC-CMs) to characterize the transcriptional impact of CFZ and to evaluate whether atorvastatin could prevent these deleterious transcriptional changes. hiPSC-CMs were treated with 1 µM CFZ, CFZ + atorvastatin, atorvastatin, or vehicle control, followed by RNA sequencing, differential expression analyses, and pathway analyses. Transcriptomic profiling revealed a marked upregulation of genes in multiple proteasome subunits, including ATPase components (*PSMC1*, *PSMC4*, *PSMC5*, *PSMC6*) and non-ATPase regulatory subunits (*PSMD1*, *PSMD2*, *PSMD12*), suggesting a strong compensatory activation of proteostasis and protein quality-control pathways in response to CFZ exposure. In addition, several of the most significantly altered genes were those implicated in cardiomyopathy and heart failure, such as *BAG3* and *FLNC*, and many heat-shock proteins, indicating the activation of cardiac stress–response pathways relevant to CFZ-associated cardiotoxicity. Atorvastatin co-treatment partially reversed a subset of CFZ-induced transcriptional changes, particularly within cholesterol biosynthesis and lipid-regulatory pathways (e.g., *ACAT2* and *ACTA1*) but did not restore the CFZ-mediated downregulation of sarcomeric genes. Together, these findings define a multifactorial signature of deleterious CFZ-induced transcriptional changes and suggest that atorvastatin may provide partial metabolic, but not structural, cardio protection.

## 1. Introduction

The proteasome inhibitor carfilzomib (CFZ) has been used as the backbone for first-line combination therapy for patients with relapsed or refractory multiple myeloma (MM), the second-most common hematologic cancer [1,2]. However, despite its effectiveness, CFZ is associated with significant cardiovascular adverse events (CVAEs), including hypertension, arrhythmias, and heart failure, with incidence estimates ranging from 8 to 18% [3]. A critical clinical implication of this cardiotoxicity is treatment interruption, which is associated with cancer recurrence [4]. Studies have shown that the early detection of and intervention for cardiotoxicity induced by other cancer therapies (i.e., anthracyclines) can improve long-term outcomes [5,6]. It is crucial to understand the underlying pathophysiological and inherent compensatory mechanisms of CFZ-related CVAEs to develop effective strategies for the early detection and management of high-risk patients [4].

Statins are a group of lipid-lowering drugs that inhibit cholesterol biosynthesis by inhibiting 3-Hydroxy-3-Methylglutaryl-Coenzyme A (HMG-CoA) reductase [7] and have been approved and widely used for over 30 years to prevent coronary artery disease and stroke [8,9]. Statins also have pleiotropic effects beyond their cholesterol-lowering properties, such as reducing vascular and systemic inflammation [10,11], decreasing the formation of reactive oxygen species (ROS) [12], and improving endothelial function and nitric oxide bioavailability [13,14]. Different studies, including the recently published STOP-CA randomized clinical trial, have demonstrated that statins can reduce cardiac functional impairment in patients with cancer treated with anthracyclines [15,16,17,18]. More importantly, a mouse study reported that atorvastatin prevented CFZ-mediated left ventricular dysfunction and molecular deficits in mice with cardiometabolic syndrome [19]. We hypothesize that atorvastatin may also protect against CFZ-related CVAE in humans. We used a human induced pluripotent stem cell-derived cardiomyocyte (hiPSC-CM) model to test this hypothesis. hiPSCs can self-renew and differentiate into various cell types, including cardiomyocytes [20,21]. The advantages of hiPSC-CMs include the perfect genetic homology between each line and their human counterpart, providing a more physiologically relevant model compared to immortalized cardiac cell lines, and their demonstrated utility for high-throughput applications [22]. hiPSC-CMs exposed to other cardiotoxic medications such as doxorubicin, kinase inhibitors, and trastuzumab were able to recapitulate the phenotypes of cardiac dysfunction, such as decreased cardiomyocyte viability and contractility, impaired mitochondrial and metabolic function, and increased reactive oxygen species (ROS) production [22,23,24]. In this study, we aimed to investigate the underlying mechanisms of CFZ-CVAEs by characterizing the transcriptional impact of CFZ treatment on hiPSC-CMs and to evaluate whether atorvastatin could prevent these deleterious transcriptional changes, using RNA sequencing analyses followed by pathway analyses.

## 2. Results

### 2.1. Validation of Pluripotent State Prior to Cardiomyocyte Differentiation

To confirm that the starting cell population had not yet undergone cardiomyocyte differentiation, we assessed the expression of key cardiac structural and mitochondrial genes in undifferentiated hiPSCs. Undifferentiated hiPSCs showed negligible expression of sarcomeric markers (*MYH6*, *MYH7*, *MYL2*, *TNNT2*) and mitochondrial regulators (*ATP2A2*, *MFN1*, *MFN2*), consistent with their pluripotent and non-cardiac phenotype (Supplementary Figure S1). The absence of these lineage-specific transcripts confirms that cardiomyocyte-enriched gene expression patterns observed in later experimental stages resulted from controlled differentiation rather than baseline expression in the parental hiPSC population. This validation step ensures that all downstream transcriptomic effects reflect true responses of differentiated cardiomyocytes rather than undifferentiated stem cells.

### 2.2. Establishing the Optimal Carfilzomib Dose for hiPSC-CM Experiments

To determine the optimal concentration of CFZ for subsequent transcriptomic and functional analyses, we performed a dose–response experiment using 0.1 µM and 1.0 µM CFZ in hiPSC-CMs, guided by previous literature reporting CFZ cardiotoxicity within this concentration range [25]. Quantitative PCR analysis of key mitochondrial and sarcomeric genes demonstrated a clear dose-dependent effect. The mitochondrial regulators *ATP2A2*, *MFN1*, and *MFN2* exhibited progressive upregulation with increasing CFZ concentrations, indicating early activation of mitochondrial stress–response programs. In contrast, core sarcomeric genes (*MYH6*, *MYH7*, and *TNNT2*) exhibited marked dose-dependent downregulation, reflecting impairment of cardiomyocyte contractile machinery. Based on these consistent transcriptional alterations and in alignment with prior studies evaluating CFZ cardiotoxicity in vitro, we selected 1.0 µM CFZ for all subsequent experiments to ensure robust and reproducible transcriptional responses while avoiding excessive cytotoxicity (Supplementary Figure S2).

### 2.3. Differentially Expressed Genes in CFZ vs. Control

**Figure 1:**
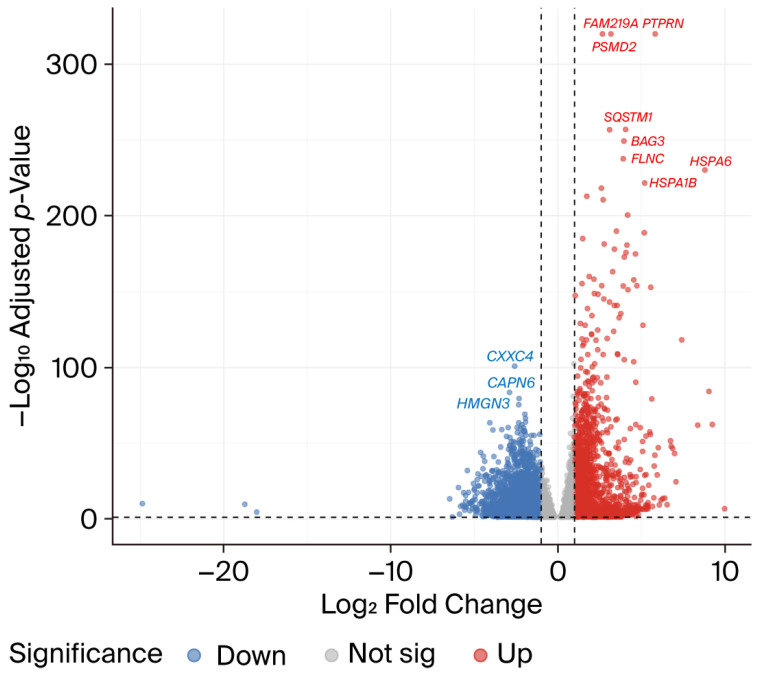
**Volcano plot of differentially expressed genes in carfilzomib-treated hiPSC-CMs compared with controls.** Volcano plot illustrating transcriptome-wide differential gene expression following 24-h treatment of hiPSC-CMs with 1 µM carfilzomib (CFZ). Each point represents a gene, plotted by log_2_ fold change (x-axis) and −log_10_(adjusted *p*-value) (y-axis). Significantly upregulated genes (log_2_FC ≥ 1 and FDR-adjusted *p* value < 0.05) are shown in red, while significantly downregulated genes (log_2_FC ≤ −1 and FDR-adjusted *p* value < 0.05) are shown in blue. Non-significant genes are visualized in gray. CFZ treatment induced substantial transcriptional remodeling, characterized by a large cluster of downregulated structural and sarcomeric genes, alongside marked upregulation of stress–response, proteostasis, and lipid-regulatory pathways. Threshold lines indicate log_2_FC = ±1 and FDR = 0.05. Differential expression analysis was performed using DESeq2.

To evaluate the impact of CFZ on global gene expression, we conducted a differential expression analysis comparing CFZ-treated hiPSC-CMs (n = 7) to controls (n = 8) using the DESeq2 framework. The dataset comprised 36,245 transcripts, ~11,000 of which had significant adjusted *p*-values. Differential expression was defined by a threshold of fold change (FC) ≥ 2 or ≤0.5 (or log2FC ≥ 1 or ≤ −1) and FDR-adjusted *p*-value < 0.05. Global gene expression profile analysis identified 6025 differentially expressed genes (DEGs), including 3871 downregulated genes (Supplementary Table S1) and 2154 upregulated genes (Supplementary Table S2), in CFZ-treated hiPSC-CMs compared to controls. A volcano plot summarizing the distribution of fold changes and statistical significance is shown in Figure 1.

Among the top 100 DEGs in hiPSC-CMs treated with CFZ compared to controls, 98 were upregulated and 2 were downregulated. The most significantly upregulated genes were the following: (1) genes in the ubiquitin proteasomal system, including proteasome 26S subunit, non-ATPase 2 (*PSMD2*) (log_2_FC = 2.67, *p*_adj_ = 2.32 × 10^−458^), protein tyrosine phosphatase, receptor type N (*PTPRN*) (log_2_FC = 5.84, *p*_adj_ = 1.14 × 10^−340^), and heat shock protein genes such as *HSPA6* (log_2_FC = 8.80, *p*_adj_ = 4.98 × 10^−231^) and *HSPA1B* (log_2_FC = 5.20, *p*_adj_ = 2.13 × 10^−222^); (2) genes in the Nrf2 pathway or nuclear receptors pathway, including sequestosome 1 (*SQSTM1*) (log_2_FC = 4.06, *p*_adj_ = 1.22 × 10^−257^), MAF basic leucine zipper transcription factor F (*MAFF*) (log_2_FC = 4.08, *p*_adj_ = 1.98 × 10^−176^), and heparin-binding EGF-liking growth factor (*HBEGF*) (log_2_FC = 3.99, *p*_adj_ = 1.78 × 10^−173^); (3) genes involved in apoptosis modulation and signaling pathways, such as BAG cochaperone 3 (*BAG3*) (log_2_FC = 3.95, *p*_adj_ = 3.64 × 10^−250^), or the MAPK signaling pathway, such as filamin-C (*FLNC*) (log_2_FC = 3.92, *p*_adj_ = 2.19 × 10^−238^) and dual specificity phosphatase 1 (*DUSP1*) (log_2_FC = 4.56, *p*_adj_ = 1.39 × 10^−158^) (Supplementary Table S2). Among the most significantly downregulated genes were CXXC finger protein 4 (*CXXC4*) (log_2_FC = −2.58, *p*_adj_ = 1.24 × 10^−101^), calpain 6 (*CAPN6*) (log_2_FC = −2.89, *p*_adj_ = 3.60 × 10^−84^), high mobility group nucleosomal binding domain 3 (*HMGN3*) (log_2_FC = −2.32, *p*_adj_ = 2.91 × 10^−80^), and fibroblast growth factor receptor 2 (*FGFR2*) (log_2_FC = −2.34, *p*_adj_ = 3.15 × 10^−76^) (Supplementary Table S1).

**Figure 2:**
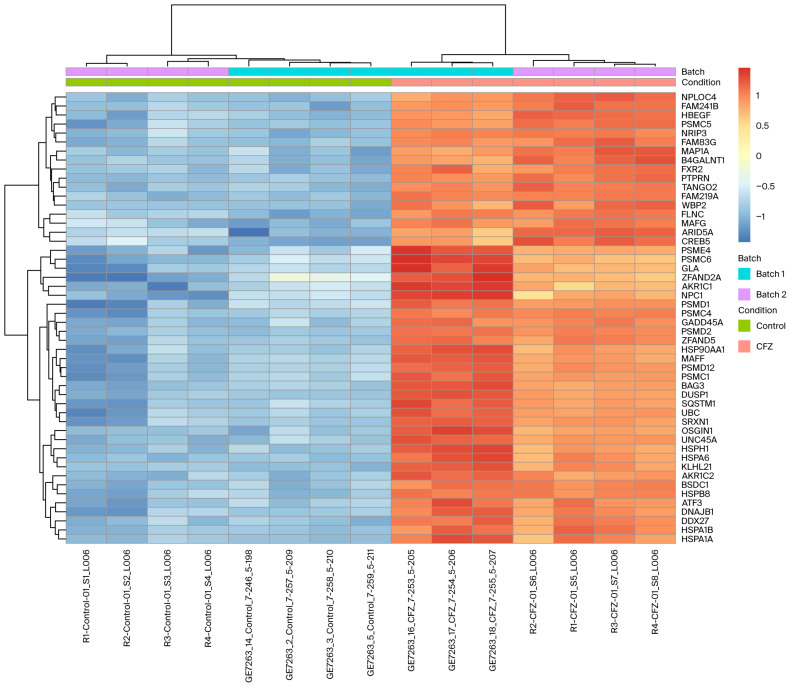
Heatmap showing the top 50 differentially expressed genes between carfilzomib-treated and control hiPSC-derived cardiomyocytes. Rows represent genes, and columns represent samples, with the expression Z-score-scaled across samples. Carfilzomib-treated cells cluster distinctly from controls and show marked upregulation of proteasome- and stress–response genes (e.g., *PSMC5*, *PSMD1*, *HSPA1A*) and downregulation of metabolic and regulatory genes. Batch annotations indicate that clustering is driven primarily by treatment conditions.

To further characterize the transcriptional response to CFZ, we generated a heatmap of the top 50 differentially expressed genes between CFZ-treated and control hiPSC-CMs (Figure 2). Unsupervised hierarchical clustering clearly separated CFZ-treated samples from controls, indicating a robust and highly consistent treatment-specific signature across biological replicates. Most of the top DEGs were strongly upregulated in CFZ-treated cells (orange/red) and relatively suppressed in controls (blue), including multiple proteasome subunits.

### 2.4. Functional Enrichment of CFZ-Induced Gene Expression Changes

Gene Ontology (GO) enrichment analysis revealed that CFZ treatment profoundly disrupts mitochondrial function, lipid metabolism, and cardiac structural pathways in hiPSC-CMs. Mitochondrial-related GO terms showed significant enrichment, including mitochondrial genome maintenance (GO:0000002; *p* = 0.0043), suggesting impaired mitochondrial DNA stability and replication, as well as pathways involved in outer mitochondrial membrane organization (GO:1901028, GO:1901030; *p* = 0.039 and 0.053, respectively) and mitochondrial protein processing (GO:0034982; *p* = 0.053), indicating altered mitochondrial turnover and heightened susceptibility to apoptosis (Supplementary Figure S3). CFZ also dysregulated multiple lipid metabolic processes, with enrichment of regulation of lipoprotein lipase activity (GO:0051004; *p* = 0.0138), negative regulation of lipoprotein lipase activity (GO:0051005; *p* = 0.05), positive regulation of cholesterol storage (GO:0010886; *p* = 0.05), and membrane lipid catabolic process (GO:0046466; *p* = 0.053), highlighting disturbances in cholesterol handling and membrane lipid remodeling (Supplementary Figure S4). In addition, cardiac-related GO terms demonstrated strong enrichment, particularly negative regulation of cardiac muscle cell differentiation (GO:2000726; *p* = 0.052), reflecting suppression of genes required for cardiomyocyte maturation, contractility, and electrical stability. Collectively, these pathway disruptions suggest that CFZ impairs mitochondrial homeostasis, lipid metabolism, and cardiac structural development, providing mechanistic insight into the reduced contractile performance and cardiotoxicity associated with CFZ treatment (Supplementary Table S3).

### 2.5. Transcriptomic Clustering Reveals Metabolic Reprogramming with Limited Sarcomeric Recovery in Cardiomyocytes Treated with CFZ + Atorvastatin

**Figure 3:**
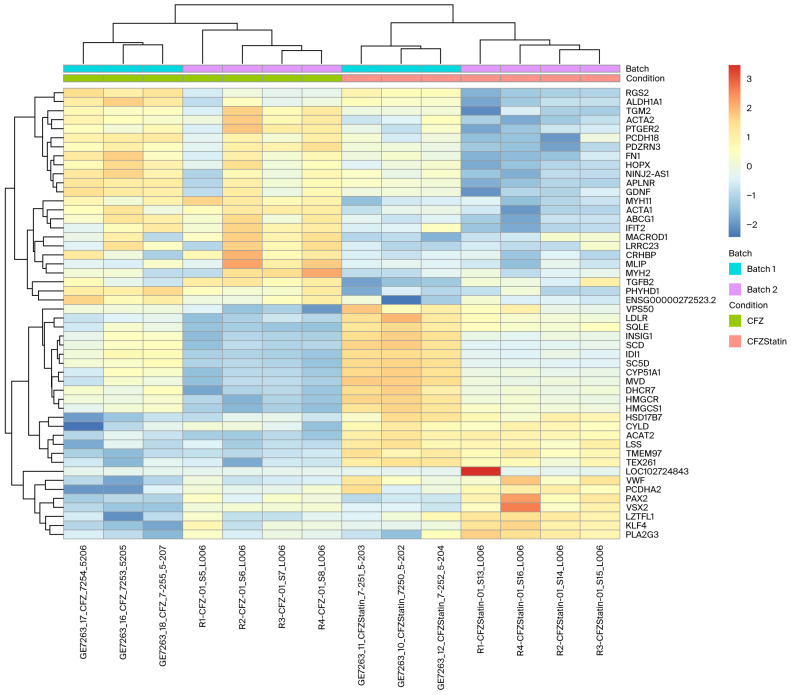
Heatmap of the top 50 differentially expressed genes comparing carfilzomib + atorvastatin treatment of hiPSC-CMs to carfilzomib alone. Samples cluster distinctly by treatment, indicating a clear transcriptional shift with atorvastatin co-treatment. Key lipid-regulatory genes (*HMGCR*, *HMGCS1*, *INSIG1*, *ACAT2*) show marked upregulation with atorvastatin, reflecting activation of cholesterol and metabolic pathways, whereas sarcomeric and contractile genes (*ACTA1*, *MYH11*, *MYLPF*) remain suppressed. This pattern suggests that atorvastatin primarily restores metabolic processes without fully rescuing carfilzomib-induced structural gene downregulation.

Unsupervised hierarchical clustering of the top 50 differentially expressed genes between CFZ + atorvastatin and CFZ-treated hiPSC-CMs revealed two distinct expression clusters corresponding to each treatment condition, indicating a strong transcriptional shift upon atorvastatin co-treatment, as shown in Figure 3 and Supplementary Table S4. Genes associated with lipid and cholesterol metabolism, including 3-hydroxy-3-methylglutaryl-CoA reductase (*HMGCR*) (log_2_FC = 0.96, *p*_adj_ = 2.19 × 10^−32^), 3-hydroxy-3-methylglutaryl-CoA synthase 1 (*HMGCS1*) (log_2_FC = 1.21, *p*_adj_ = 2.21 × 10^−17^), Low Density Lipoprotein Receptor (*LDLR*) (log_2_FC = 0.74, *p*_adj_ = 3.57 × 10^−9^), acetyl-CoA acetyltransferase 2 (*ACAT2*) (log_2_FC = 1.32, *p*_adj_ = 2.57 × 10^−8^), and insulin induced gene 1 (*INSIG1*) (log_2_FC = 1.26, *p*_adj_ = 4.96 × 10^−8^), were upregulated in the CFZ + atorvastatin-treated hiPSC-CMs compared to the cells treated with CFZ alone, consistent with reactivation of the mevalonate and cholesterol biosynthesis pathways. In contrast, sarcomeric and contractile genes such as actin alpha 1 (*ACTA1*) (log_2_FC = −1.95, *p*_adj_ = 7.89 × 10^−7^), myosin heavy chain 11 (*MYH11*) (log_2_FC = −1.96, *p*_adj_ = 0.0026), and myosin heavy chain (*MYH2*) (log_2_FC = −3.63, *p*_adj_ = 0.014) remained suppressed, suggesting that atorvastatin primarily restored metabolic pathways rather than structural cardiac programs. Additionally, modest upregulation of stress–response and signaling genes such as von Willebrand factor (*VWF*) (log_2_FC = 1.35, *p*_adj_ = 3.67 × 10^−5^) and CYLD lysine 63 deubiquitinate (*CYLD*) (log_2_FC = 0.37, *p*_adj_ = 0.0037), and partial reductions in inflammatory mediators such as interferon induced protein with tetratricopeptide repeats 2 (*IFIT2*) (log_2_FC = −1.23, *p*_adj_ = 0.011) and corticotropin releasing hormone binding protein (*CRHBP*) (log_2_FC = −1.39, *p*_adj_ = 0.0072) were observed, reflecting a mixed protective adaptation at the transcriptional level (Supplementary Table S4).

### 2.6. Atorvastatin Reverses a Subset of CFZ-Induced Transcriptional Alterations

**Figure 4:**
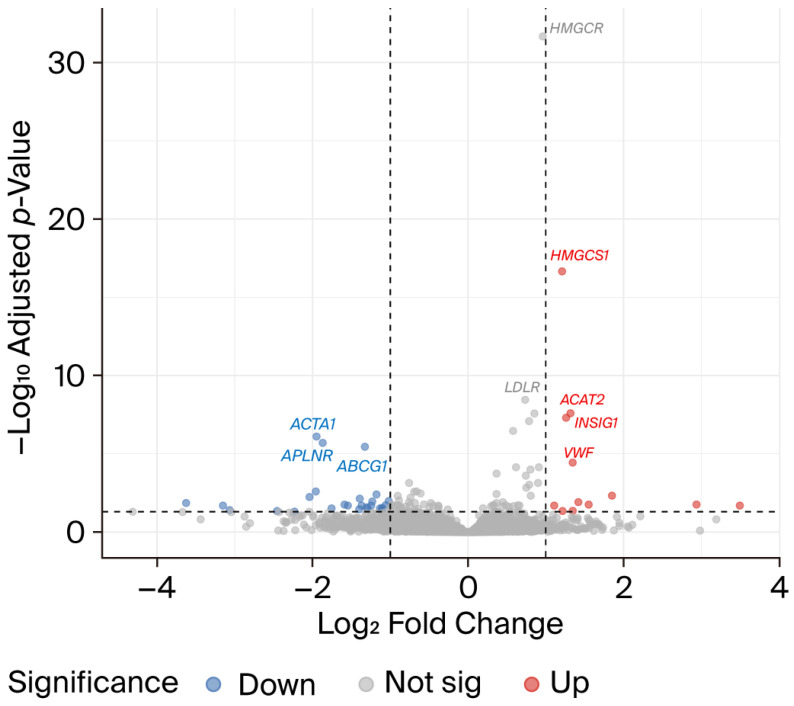
Volcano plot highlighting differentially expressed genes between carfilzomib + atorvastatin and carfilzomib-treated hiPSC-CMs compared with carfilzomib vs. controls.

**Figure 5:**
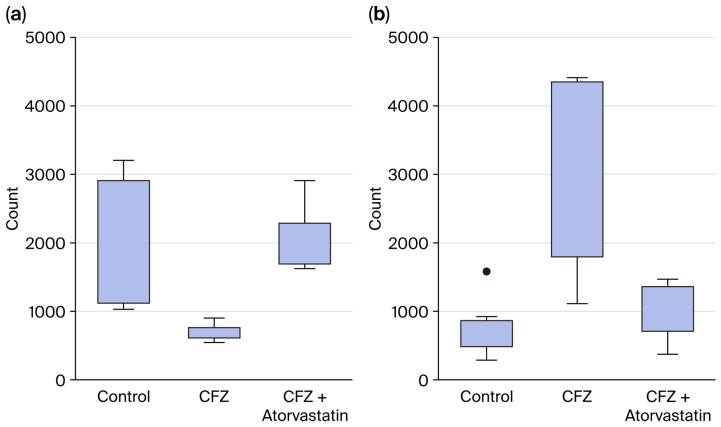
Expression of top genes treated with control, carfilzomib, or carfilzomib + atorvastatin. (**a**) *ACAT2* gene; (**b**) *ACTA1* gene. CFZ: carfilzomib. CFZ: carfilzomib. In box plots, the boxes contain the middle 50% of the data with the first quartile on the bottom and the third quartile on the top; the whiskers represent 1.5 times above or below the interquartile range. The dot denotes outlier, i.e., an observation that falls outside the range of the whiskers.

To investigate whether atorvastatin mitigates the molecular effects of CFZ treatment, we performed a comparative differential expression analysis between the CFZ vs. control and CFZ + atorvastatin vs. CFZ conditions, as shown in the volcano plot Figure 4. A small subset of genes (n = 12) exhibited significant changes in opposite directions across the two contrasts, indicating directional reversal of CFZ-induced transcriptional effects by atorvastatin co-treatment (Table 1). Among these, eight genes (*ACAT2*, *PAX2*, *PLA2G3*, *VSX2*, *PCDHA2*, *DERL3*, *NDST4*, and *TMEM178B*) were downregulated by CFZ but upregulated upon atorvastatin co-treatment. These genes are functionally enriched in lipid metabolic regulation (*ACAT2*, *PLA2G3*), endoplasmic reticulum (ER) stress and protein quality control (*DERL3*), and cellular signaling and transcriptional regulation (*PAX2*, *VSX2*, *TMEM178B*), suggesting that atorvastatin may restore metabolic and ER homeostasis disrupted by CFZ exposure. Conversely, four genes (*ACTA1*, *CRHBP*, *IFIT2*, and *PTGER2*) were upregulated by CFZ but suppressed by atorvastatin co-treatment. These genes are associated with sarcomeric contractile function (*ACTA1*), stress–response signaling (*CRHBP*, *IFIT2*), and prostaglandin receptor-mediated inflammation (*PTGER2*). Their downregulation following atorvastatin treatment indicates a potential attenuation of CFZ-induced cellular stress and contractile dysregulation. The gene expression levels of *ACAT2* and *ACTA1* are demonstrated in Figure 5 as examples. Collectively, these findings demonstrate that, while most CFZ-responsive genes remain unaltered by atorvastatin, a discrete subset shows reversal of CFZ-induced expression patterns, supporting the notion that atorvastatin may exert protective transcriptional effects against CFZ-mediated cardiotoxicity, particularly through pathways involved in lipid metabolism, ER stress resolution, and cytoskeletal maintenance.

### 2.7. Validation of RNA-Seq Findings by qPCR

To evaluate the effects of CFZ and atorvastatin on cardiac structural and mitochondrial gene expression, we performed qPCR analysis of *ATP2A2*, *MFN1*, *MFN2*, *MYH6*, *MYH7*, *MYL2*, and *TNNT2* across four experimental conditions: control, CFZ alone, atorvastatin alone, and CFZ + atorvastatin. CFZ treatment markedly suppressed the expression of key sarcomeric genes (*MYH6*, *MYH7*, *MYL2*, *TNNT2*), which was consistent with the impaired contractile gene programs observed in our RNA-seq analysis. Atorvastatin alone had a minimal impact on these cardiac markers, indicating limited transcriptional influence in atorvastatin-treated cardiomyocytes. Importantly, co-treatment with CFZ + atorvastatin did not restore sarcomeric gene expression, suggesting that atorvastatin is unable to rescue CFZ-induced transcriptional suppression of structural genes. In contrast, mitochondrial and calcium-handling genes (*ATP2A2*, *MFN1*, *MFN2*) showed modest increases with atorvastatin and partial normalization in the CFZ + atorvastatin condition, indicating that statins may preferentially modulate metabolic pathways rather than structural cardiac programs. Together, these qPCR findings validate our transcriptomic results, demonstrating that atorvastatin exerts a metabolic protective effect but does not reverse CFZ-induced downregulation of cardiac contractile genes (Supplementary Figure S5).

## 3. Discussion

In this study, we investigated the transcriptomic effects of CFZ on hiPSC-CMs and assessed whether atorvastatin could mitigate deleterious CFZ-induced transcriptional alterations. We observed striking transcriptional changes induced by CFZ that are consistent with proteasome inhibition. More importantly, our results demonstrated that CFZ profoundly disrupts cardiomyocyte homeostasis by altering pathways essential for cardiac metabolism, lipid regulation, mitochondrial function, oxidative stress regulation, apoptosis, and sarcomeric integrity, which are mechanisms that closely mirror the cardiovascular adverse events observed clinically, including heart failure, arrhythmias, and cardiomyopathy. We also observed that atorvastatin co-treatment partially reversed a subset of CFZ-induced transcriptional changes, particularly within cholesterol biosynthesis and lipid-regulatory pathways (e.g., *ACAT2* and *ACTA1*) but did not restore CFZ-mediated downregulation of sarcomeric genes.

The ubiquitin proteasome pathway is the major cellular proteolytic pathway for maintaining cellular proteome stability and proper cellular function and viability [26]. In patients with MM, malignant plasma cells produce excessive amounts of immunoglobulins, inducing significant proteotoxic stress, and are highly dependent on proteasome activity to survive, making them sensitive to proteasome inhibition [4]. By selectively and irreversibly binding the β5 subunit of the 26S proteasome and blocking its chymotrypsin-like activity that normally degrades poly-ubiquitinated proteins [27], CFZ can delay proliferation and induce cell-cycle arrest and apoptosis in malignant plasma cells in the bone marrow of patients with MM [28]. Terminally differentiated cardiomyocytes are post-mitotic cells and thus especially sensitive to proteasome inhibition [29]. In addition, the high metabolic demand of the heart increases the burden of proteostasis. Therefore, proteasome inhibition may be deleterious for the heart [4].

In our study of hiPSC-CMs, among the most prominent transcriptomic changes induced by CFZ treatment were the significant upregulation of multiple proteasome subunits, including ATPase components (*PSMC1*, *PSMC4*, *PSMC5*, *PSMC6*) and non-ATPase regulatory subunits (*PSMD1*, *PSMD2*, *PSMD12*), which indicates a robust compensatory response to proteasome inhibition. These genes are essential for substrate recognition, unfolding, and degradation, suggesting that CFZ-treated hiPSC-CMs activate proteostasis and protein quality-control pathways to counteract the accumulation of misfolded proteins [30]. Among other notable upregulated genes were *SQSTM1*, *BAG3*, *FLNC*, *HSPA6*, and *HSPA1B*. *SQSTM1* acts as a ubiquitin-binding scaffold that redirects the overload of poly-ubiquitinated proteins toward autophagy [31]. *BAG3*, together with the stress-inducible Hsp70 isoforms (*HSPA6* and *HSPA1B*), forms a chaperone-assisted selective autophagy complex [32]. Our results confirmed that CFZ’s blockage of the proteasome elicits a multilayered adaptive network to rebuild proteasome capacity or reroute the surplus of ubiquitinated proteins into autophagy. Importantly, several of these genes have been directly implicated in cardiovascular disease and heart failure and have been identified in a previous hiPSC-CM study [25], including *BAG3* [33,34,35] and multiple heat-shock proteins (*HSPA1A/B/L*, *HSPH1*, *HSPB8*) [36]. These genes mediate essential processes such as sarcomere maintenance, mitochondrial respiration, oxidative stress defense, apoptosis, and proteostasis—all of which are disrupted in CFZ-induced cardiotoxicity. Their consistent upregulation in CFZ-treated samples further confirms the activation of cardiac stress–response pathways known to contribute to heart failure. More importantly, genetic variants in the *BAG3* and *FLNC* genes have been associated with cardiomyopathies in both familial and population studies. Multiple rare variants in the *BAG3* gene have been identified as causative of dilated cardiomyopathy in different cohorts [37,38,39]. Truncating *FLNC* mutations are associated with high-risk dilated and arrhythmogenic cardiomyopathies [40] and familial restrictive cardiomyopathy [41]. A novel combination of a *BAG3* (c.601G.A; p.Gly204Arg) and *FLNC* (c.5707G>A; p.Glu1903Lys) genetic variant was found to express a pediatric restrictive cardiomyopathy phenotype [42]. In light of the literature, our findings suggest that there might be shared pathophysiological mechanisms between CFZ-induced cardiotoxicity and genetic cardiomyopathies.

CFZ-treated hiPSC-CMs also exhibited extensive downregulation of genes critical for cardiac structure and contractility, including *MYH6*, *MYH7*, *MYL2*, and *TNNT2* [43]. Suppression of these sarcomeric genes is strongly associated with impaired contraction, reduced force generation, and cardiomyocyte differentiation—all features reported in patients who develop CFZ-associated systolic dysfunction and heart failure [3,44]. These transcriptomic findings align with clinical and preclinical studies demonstrating impaired cardiac output, altered calcium handling, and structural remodeling after CFZ exposure [45,46].

In addition to structural gene suppression, CFZ significantly altered mitochondrial pathways. The enrichment of mitochondrial genome maintenance, outer membrane organization, and mitochondrial protein processing pathways suggests that CFZ disrupts mitochondrial homeostasis [25,47,48]. Mitochondrial instability is directly linked to reduced ATP production [49], bioenergetic failure [50,51,52], and increased ROS, which are established drivers of heart failure pathogenesis [53]. Oxidative stress emerged as a key feature of the CFZ transcriptomic signature. Genes involved in oxidative phosphorylation, oxidative RNA/DNA demethylation, and oxidative stress-induced apoptosis were significantly enriched [54,55]. These pathways reflect mitochondrial-derived ROS accumulation and redox imbalance—mechanisms known to contribute to cardiomyocyte apoptosis and left ventricular dysfunction [53]. These findings are consistent with prior studies showing that CFZ increases oxidative stress markers, reduces mitochondrial membrane potential, and triggers cardiomyocyte injury [53,56]. Apoptotic pathways were also robustly represented among CFZ-regulated genes. Enrichment of intrinsic apoptotic signaling, caspase activation, and execution-phase apoptosis supports the idea that CFZ induces programmed cell death in cardiomyocytes [57,58]. Apoptosis is a hallmark of many forms of chemotherapy-induced cardiotoxicity and correlates strongly with clinical cardiac dysfunction [53,59,60].

Due to the beneficial effects of statins demonstrated in preventing cardiotoxicity induced by other cardiotoxic drugs [15,16,17,18], we also evaluated whether atorvastatin could prevent the deleterious CFZ-induced transcriptional changes in hiPSC-CMs. Atorvastatin co-treatment upregulated genes involved in cholesterol biosynthesis and lipid homeostasis, including *HMGCR*, *HMGCS1* and *LDLR*, consistent with compensatory up-regulations of these genes in a classic feedback response. Notably, *ACAT2* was markedly suppressed by CFZ and but was restored by atorvastatin co-treatment. However, atorvastatin did not rescue CFZ-induced downregulation of sarcomeric genes, such as *MYH6*, *MYH7*, *MYL2*, and *TNNT2*. This indicates that, while atorvastatin may correct metabolic disturbances, it does not reverse the structural deterioration or contractile gene suppression that underlies CFZ-induced systolic dysfunction. This difference underscores the complexity of CFZ cardiotoxicity and suggests that atorvastatin therapy alone may offer partial metabolic protection but not full preservation of cardiac function.

Collectively, our findings support a multifactorial model of CFZ-induced cardiotoxicity in which several interconnected mechanisms contribute to cardiomyocyte injury. CFZ exposure disrupts mitochondrial function, leading to impaired energy production and increased vulnerability to metabolic stress. This mitochondrial instability is accompanied by excessive oxidative stress and accumulation of ROS, which further damages cellular components and exacerbates redox imbalance. In parallel, CFZ activates intrinsic apoptotic pathways, promoting programmed cell death and loss of viable cardiomyocytes. These molecular disturbances are compounded by the marked suppression of key structural and contractile genes essential for maintaining sarcomere integrity and cardiac mechanical function. Additionally, CFZ alters lipid and cholesterol metabolism, a process fundamental for membrane stability, mitochondrial health, and cellular signaling. Together, these molecular signatures closely mirror the clinical phenotypes observed in CFZ-treated patients, including reduced left ventricular ejection fraction, diminished contractile reserve, arrhythmia, and heart failure. Notably, the partial restoration of metabolic pathways by atorvastatin—particularly those related to cholesterol regulation, ER stress mitigation, and mitochondrial stabilization—suggests a promising, though incomplete, cardioprotective potential that warrants further investigation.

It is important to acknowledge some limitations. This study used a single hiPSC line and focused on acute (24 h) drug exposure, which may not fully capture chronic or cumulative toxicity. Additional investigations using electrophysiology, contractility, calcium imaging, and mitochondrial respiration are needed to confirm the functional consequences of the transcriptomic changes observed here. Further studies should evaluate dose- and timing-dependent atorvastatin effects, compare multiple statins, and investigate combination therapies targeting both metabolic and structural injury mechanisms. Another limitation of the current study is that we focused on CFZ-induced gene expression changes without evaluating the changes at the protein or metabolomic level. Integrative analysis incorporating proteomic and metabolomic profiles would be the focus of our future investigations.

## 4. Materials and Methods

### 4.1. Human-Induced Pluripotent Stem Cell-Derived Cardiomyocyte Differentiation

hiPSCs (line SCTi003-A from StemCell Technologies, Cambridge, MA, USA) were differentiated toward cardiomyocytes using small molecules or growth factors based on the manufacturer’s protocol. Briefly, hiPSCs were cultured on Matrigel^®^-coated plates in mTeSR™ Plus. For dissociation, cells were washed with D-PBS, treated with Gentle Cell Dissociation Reagent for 8–10 min at 37 °C, and resuspended in mTeSR™ medium with 10 μM Y-27632. Following centrifugation (300× *g*, 5 min), cells were seeded at a density of 3.58*10^5^ cells/well onto Matrigel^®^-coated plates. After 24 h of incubation, the medium was refreshed, and cells were cultured to >95% confluency before differentiation. Cardiomyocyte differentiation was initiated once the hiPSCs reached >95% confluency. On Day 0, the culture medium was replaced with the STEMdiff™ Ventricular Cardiomyocyte Differentiation Medium A, supplemented with Matrigel^®^ (1:100). The medium was changed every two days, using Medium B on Day 2, Medium C on Days 4 and 6, and STEMdiff™ Cardiomyocyte Maintenance Medium from Day 8 onward. Beating cardiomyocytes were observed by Day 8, and cells were ready for assays by Day 15. For long-term maintenance, the medium was changed every 2 days using Cardiomyocyte Maintenance Medium.

### 4.2. Drug Preparation and Treatment

The stock solution of CFZ (Selleck Chemicals, Houston, TX, USA) was 10 mmol/L. Based on the pharmacokinetic profile of CFZ, the peak plasma concentration is 5.88 µmol/L. Following the initial testing at 1, 2, 10, and 20 µmol/L, a dose range of 0.01 to 10 µmol/L was established [25]. A concentration of 1 µmol/L for CFZ was determined for all the following experiments. On the day of each experiment, CFZ was diluted in STEMdiff™ Cardiomyocyte Maintenance Medium to achieve a 2× final concentration and kept on ice in the dark. Dimethyl sulfoxide (DMSO) at 0.2% (*v*/*v*), equivalent to the highest drug concentration, was used as a vehicle control. Atorvastatin (Selleck Chemicals, Houston, TX, USA) at 10 µmol/L was evaluated as a potential cardioprotective agent against deleterious CFZ-induced transcriptional changes, with its stock solution (10 mmol/L) prepared similarly to CFZ.

hiPSC-CMs were treated on Day 15 of differentiation with the following conditions: (1) CFZ alone (1 µM), (2) atorvastatin alone (1 µM), (3) CFZ + atorvastatin (same concentration), and (4) untreated controls. All treatments were adjusted to contain equivalent concentrations of DMSO (0.2% *v*/*v*). Cells were incubated with drugs for 24 h, after which total RNA was harvested on Day 16 using the RNeasy Fibrous Tissue Mini Kit (Qiagen, Germantown, MD, USA) according to the manufacturer’s instructions.

### 4.3. Quantitative Reverse Transcription–Polymerase Chain Reaction

Total RNA was extracted using the Zymo total RNA mini kit (Zymo, Irvine, CA, USA) according to the manufacturer’s instructions. One microgram of total RNA was used for complementary DNA synthesis using the High-Capacity RNA-to-cDNA kit (Applied Biosystems, Foster City, CA, USA), and the reaction mixture was incubated using a VeritiPro thermal cycler (Applied Biosystems, Foster City, California, USA) as follows: 37 °C for 1 h and 95 °C for 5 min. The reaction mixture was further diluted, and 10 ng of complementary DNA as the template was subjected to quantitative reverse transcription–polymerase chain reaction (RT-PCR), which was performed in triplicate for each gene using a Taqman Fast Advanced Master Mix (Applied Biosystems, Foster City, CA, USA). The real-time polymerase chain reaction (PCR) conditions included an initial denaturation step at 95 °C for 20 s, and 40 cycles of 2 steps with 1 s of denaturation at 95 °C followed by 20 s of annealing at 60 °C, using the QuantStudio™ 12K Flex Real-Time PCR System (Applied Biosystems, Foster City, CA, USA). The messenger RNA levels of the genes examined were normalized to *GAPDH*. The primers used for qPCR are listed in Supplementary Table S5.

### 4.4. RNA Library and RNA Sequencing

Total RNA was extracted from hiPSC-CMs exposed to different treatment conditions—control (n = 8), CFZ (n = 7), atorvastatin (n = 5), and CFZ + atorvastatin (n = 7)—using the RNeasy Fibrous Tissue Mini Kit (Qiagen, Germantown, MD, USA). The RNA integrity and quality were confirmed prior to library preparation and sequencing. The sample quality was assessed using Agilent TapeStation 4200 (Agilent Technologies, Inc., Santa Clara, CA, USA). One hundred nanograms of total RNA was used for library construction using the NEB Ultra II Directional RNA-seq library preparation kit for Illumina according to the manufacturer’s protocol. First, mRNA was isolated from 100 ng of total RNA using the NEBNext Poly(A) mRNA Magnetic Isolation Module (New England Biolabs, catalog #E7490, Ipswich, MA, USA). This was followed by RNA library construction with the NEBNext Ultra II Directional RNA Library Prep Kit (New England Biolabs, catalog #E7760, Ipswich, MA, USA) according to the manufacturer’s user guide. Briefly, RNA was fragmented in NEBNext First Strand Synthesis Buffer under incubation at 94 °C for the desired time. This step was followed by first-strand cDNA synthesis using reverse transcriptase and random hexamer primers. ds-cDNA was synthesized using the 2nd strand master mix provided in the kit, followed by end-repair and adaptor ligation. At this point, Illumina adaptors were ligated to the sample. Finally, each library (uniquely barcoded) was enriched through 12 cycles of amplification and purified using Agencourt AMPure beads (Beckman Coulter, catalog #A63881, Brea, CA, USA). A total of 27 barcoded libraries were sized on the TapeStation 4200 and quantified with the Qubit^®^ 2.0 Fluorometer (Thermo Fisher Scientific, Carlsbad, CA, USA). Finally, these 27 individual libraries were pooled in equimolar concentration and sequenced on a NovaSeq X 10B flow cell with a run of 2 × 150 cycles. One lane generated 1.25 billion paired-end reads with an average Q30% ≥ 92.5% and Cluster PF = 85.4%. FastQ files were generated using the BCL2fastQ function in the Illumina BaseSpace portal. One NovaSeq X 2 × 150-cycle lane resulted in an average of 50 million demultiplexed, paired-end reads when sequencing a pool of 27 samples. RNA-seq libraries were constructed at the University of Florida Interdisciplinary Center for Biotechnology Research (ICBR) Gene Expression Core (https://biotech.ufl.edu/gene-expression-genotyping/, RRID:SCR_019145) and sequenced at the UF ICBR NextGen Sequencing Core using the NovaSeq X Sequencer (Illumina, Inc, San Diego, CA, USA). Principal component analysis (PCA) was performed on variance-stabilized transformed (VST) expression data to visualize sample clustering. The PCA plot revealed that samples segregated along the second principal component based on sequencing batch, independent of treatment conditions. We performed these experiments in two different batches, and our analysis confirmed the presence of a batch effect. To mitigate its influence on downstream differential expression analysis, batch information was incorporated as a covariate in the DESeq2 design formula (batch + condition), a widely accepted strategy for adjusting for unwanted variation [61,62].

### 4.5. RNA-Seq Analysis and Differential Expression Analysis

Transcriptomic data was processed through a standardized RNA-seq analysis pipeline. The raw sequencing reads underwent quality control using FastQC, and adapter trimming with Trim Galore. Transcript-level quantification was performed using Salmon [63], a lightweight and accurate tool for transcript abundance estimation. The quantified transcripts were aggregated to gene-level counts using tximport [64] in R, with reference to the GENCODE human gene annotation database. Differential expression analysis was conducted using DESeq2 [62], incorporating batch effects and treatment conditions in the design formula. Pairwise comparisons were performed between treatment groups to identify differentially expressed genes (DEGs), with significance defined as an adjusted *p*-value < 0.05 (Benjamini–Hochberg false discovery (FDR) correction) and an absolute log2 fold change (FC) > 1. The results were visualized using volcano plots and MA plots generated with ggplot2 [65], and heatmaps of the top DEGs created using pheatmap. Gene identifiers were mapped to gene symbols using biomaRt [66]. Functional enrichment analyses for biological processes such as apoptosis, oxidative stress, cardiotoxicity, lipid metabolism, and mitochondrial pathways were performed using the enrichR package [67], leveraging curated pathway databases.

## 5. Conclusions

In this hiPSC-CM study, we found that CFZ induces extensive transcriptomic remodeling consistent with clinically observed cardiovascular toxicity, driven by mitochondrial dysfunction, oxidative stress, apoptosis, and repression of contractile genes. Atorvastatin partially mitigates CFZ-induced metabolic disturbances—especially cholesterol-related pathways—but does not reverse structural gene downregulation. These findings underscore the need for multi-target cardioprotective strategies and support further evaluation of statins as a potential cardioprotective agent in reducing CFZ-associated cardiac risk.

## Figures and Tables

**Table 1:** Differentially expressed genes reversed by atorvastatin in CFZ-treated hiPSC-CMs.

Gene Symbol	Gene Name	Gene ID	CFZ vs. Controls		CFZ + Atorvastatin vs. CFZ	
			Log_2_ Fold Change	*p*_adj_	Log_2_ Fold Change	*p*_adj_
*ACAT2*	acetyl-CoA acetyltransferase 2	ENSG00000120437	−1.20	7.53 × 10^−10^	1.32	2.57 × 10^−8^
*ACTA1*	actin alpha 1, skeletal muscle	ENSG00000143632	2.31	1.68 × 10^−13^	−1.95	7.89 × 10^−7^
*PAX2*	paired box 2	ENSG00000075891	−4.57	5.45 × 10^−29^	1.85	0.0047
*CRHBP*	corticotropin releasing hormone binding protein	ENSG00000145708	1.71	1.79 × 10^−7^	−1.39	0.0072
*IFIT2*	interferon induced protein with tetratricopeptide repeats 2	ENSG00000119922	1.20	7.15 × 10^−5^	−1.23	0.011
*PLA2G3*	phospholipase A2 group III	ENSG00000100078	−1.97	1.06 × 10^−8^	1.42	0.012
*VSX2*	visual system homeobox 2	ENSG00000119614	−4.69	1.23 × 10^−10^	2.94	0.017
*PCDHA2*	protocadherin alpha 2	ENSG00000204969	−1.20	0.0036	1.55	0.018
*PTGER2*	prostaglandin E receptor 2 (EP2)	ENSG00000125384	1.02	0.0148	−1.59	0.018
*DERL3*	derlin 3	ENSG00000099958	−2.82	3.01 × 10^−25^	1.11	0.020
*NDST4*	N-deacetylase/N-sulfotransferase 4	ENSG00000138653	−6.47	4.64 × 10^−14^	3.50	0.020
*TMEM178B*	transmembrane protein 178B	ENSG00000261115	−2.28	4.35 × 10^−12^	1.22	0.045

## Data Availability

The original contributions presented in this study are included in the article/Supplementary Materials. Further inquiries can be directed to the corresponding author.

## Supplementary Materials

The following supporting information can be downloaded at https://www.mdpi.com/article/10.3390/ijms27031358/s1.

## Abbreviations

The following abbreviations are used in this manuscript:

CFZ	Carfilzomib
CVAE	Cardiovascular adverse event
hiPSC-CMs	Human induced pluripotent stem cell-derived cardiomyocytes
ROS	Reactive oxygen species
MM	Multiple myeloma
