# Supplementary material for: Atorvastatin Protects Against Deleterious Carfilzomib-Induced Transcriptional Changes in Human Induced Pluripotent Stem Cell-Derived Cardiomyocytes
Source: Int J Mol Sci. 2026 Jan 29;27(3):1358. doi: 10.3390/ijms27031358 (PMC12898222; doi:10.3390/ijms27031358)

Supplementary Figures

Supplementary Figure S1. qPCR analysis showing minimal expression of cardiac structural and mitochondrial genes (ATP2A2, MFN1, MFN2, MYH6, MYH7, MYL2, TNNT2) in undifferentiated iPSCs, confirming absence of spontaneous cardiomyocyte differentiation.

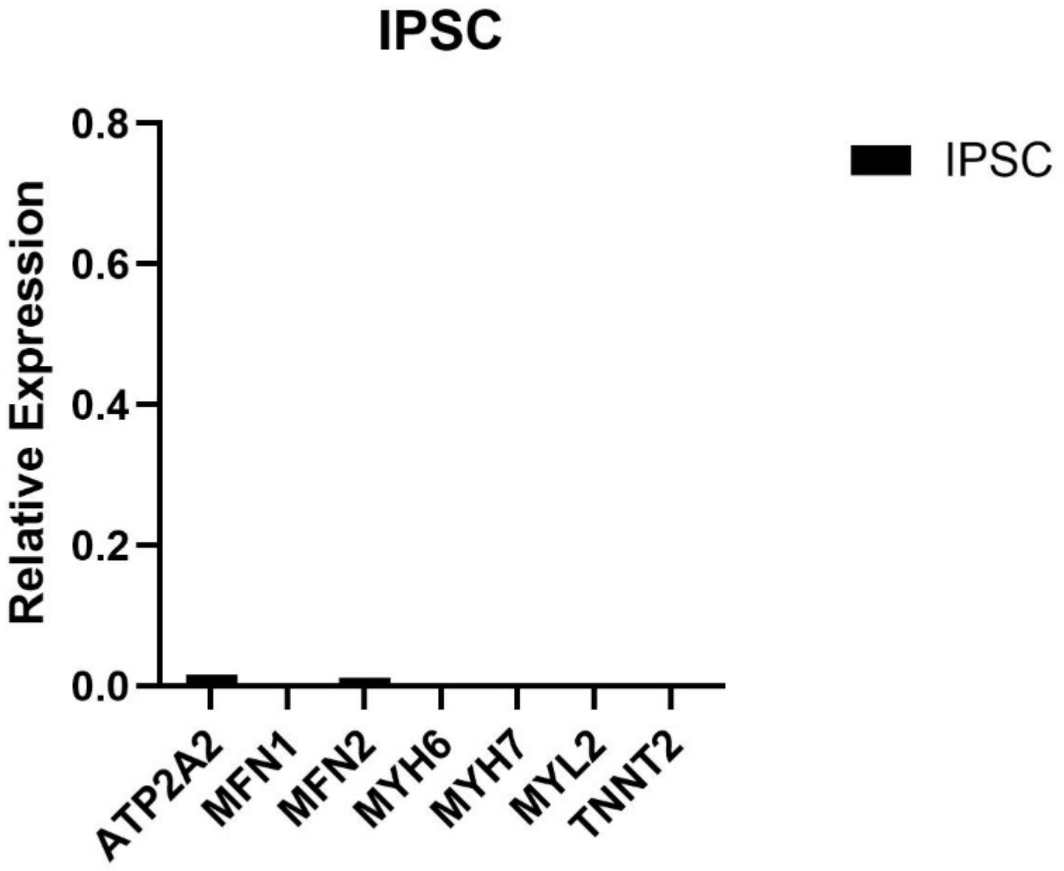

Supplementary Figure S2. Expression of mitochondrial (ATP2A2, MFN1, MFN2) and sarcomeric genes (MYH6, MYH7, MYL2, TNNT2) across DMSO, 0.1 µM CFZ, and 1.0 µM CFZ. Higher CFZ dose shows stronger downregulation of contractile genes and upregulation of mitochondrial stress markers.

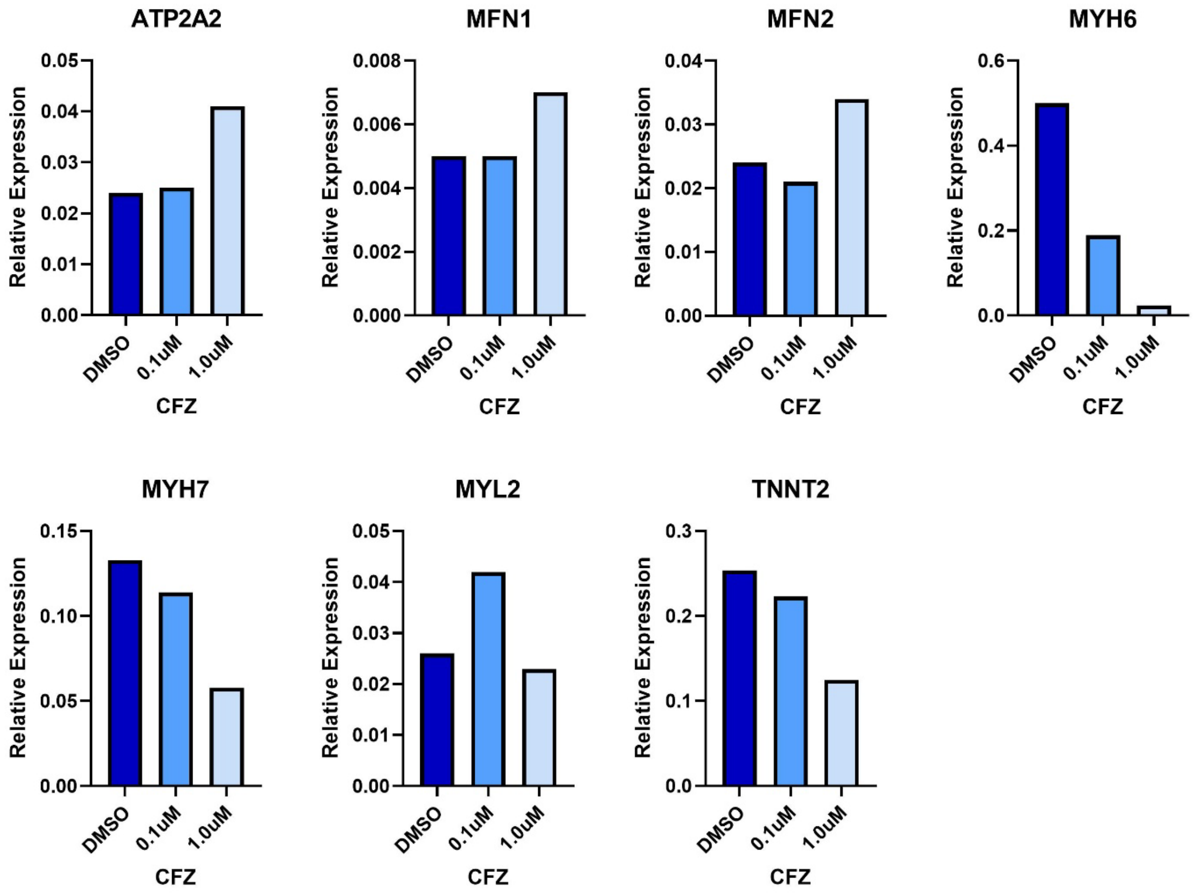

Supplementary Figure S3. GO analysis showing enrichment of mitochondrial genome maintenance, outer membrane organization, and apoptotic membrane permeabilization pathways in CFZ-treated hiPSC-CMs.

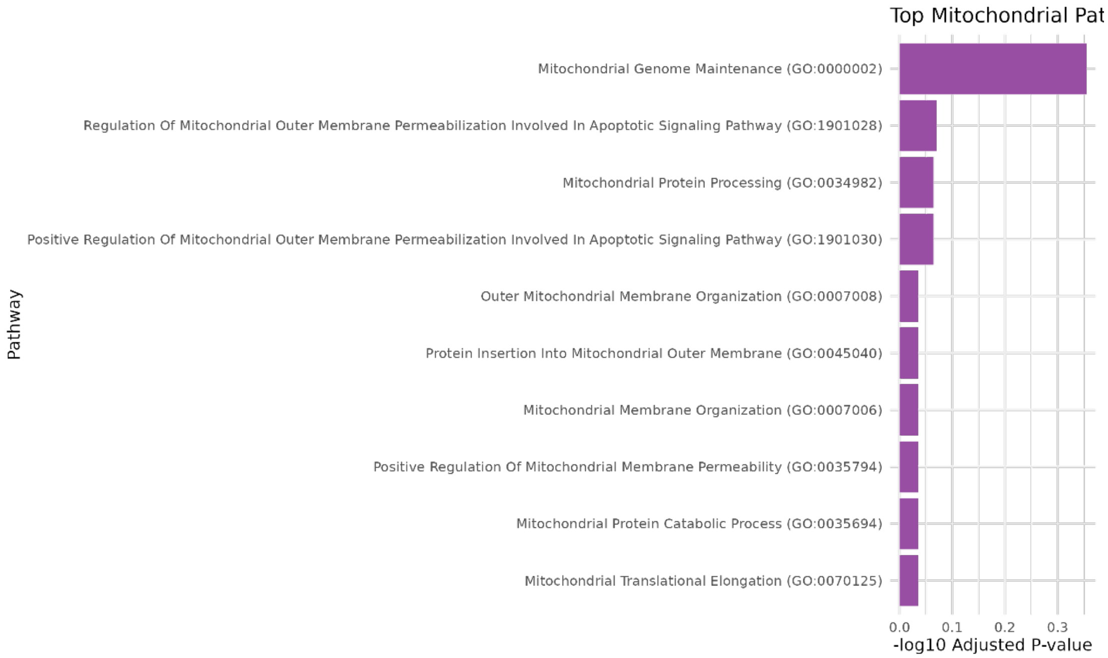

Supplementary Figure S4. CFZ significantly alters lipid-associated processes, including regulation of lipoprotein lipase activity, cholesterol storage, membrane lipid catabolism, and glycerolipid biosynthesis, indicating broad lipid metabolic disruption.

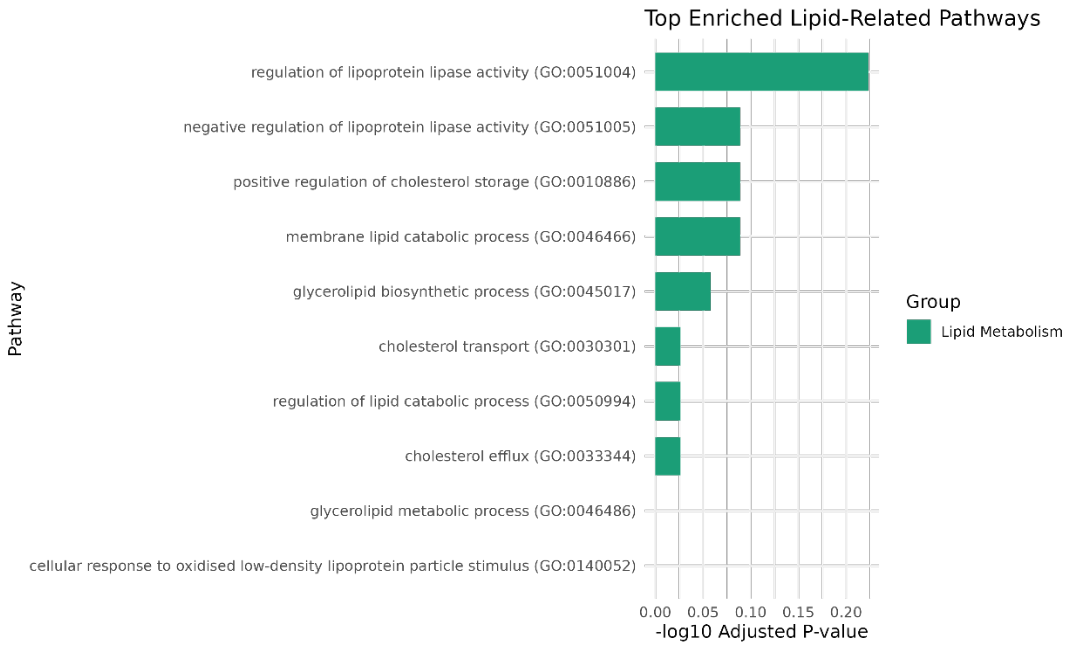

Supplementary Figure S5. Relative expression of mitochondrial (ATP2A2, MFN1, MFN2) and sarcomeric (MYH6, MYH7, MYL2, TNNT2) genes in hiPSC-CMs treated with Control, CFZ, Atorvastatin, or CFZ + Atorvastatin. CFZ markedly suppresses sarcomeric gene expression, while atorvastatin alone shows minimal effect. Co-treatment (CFZ + Atorvastatin) does not restore sarcomeric gene expression but partially normalizes mitochondrial gene levels, consistent with RNA-seq findings.

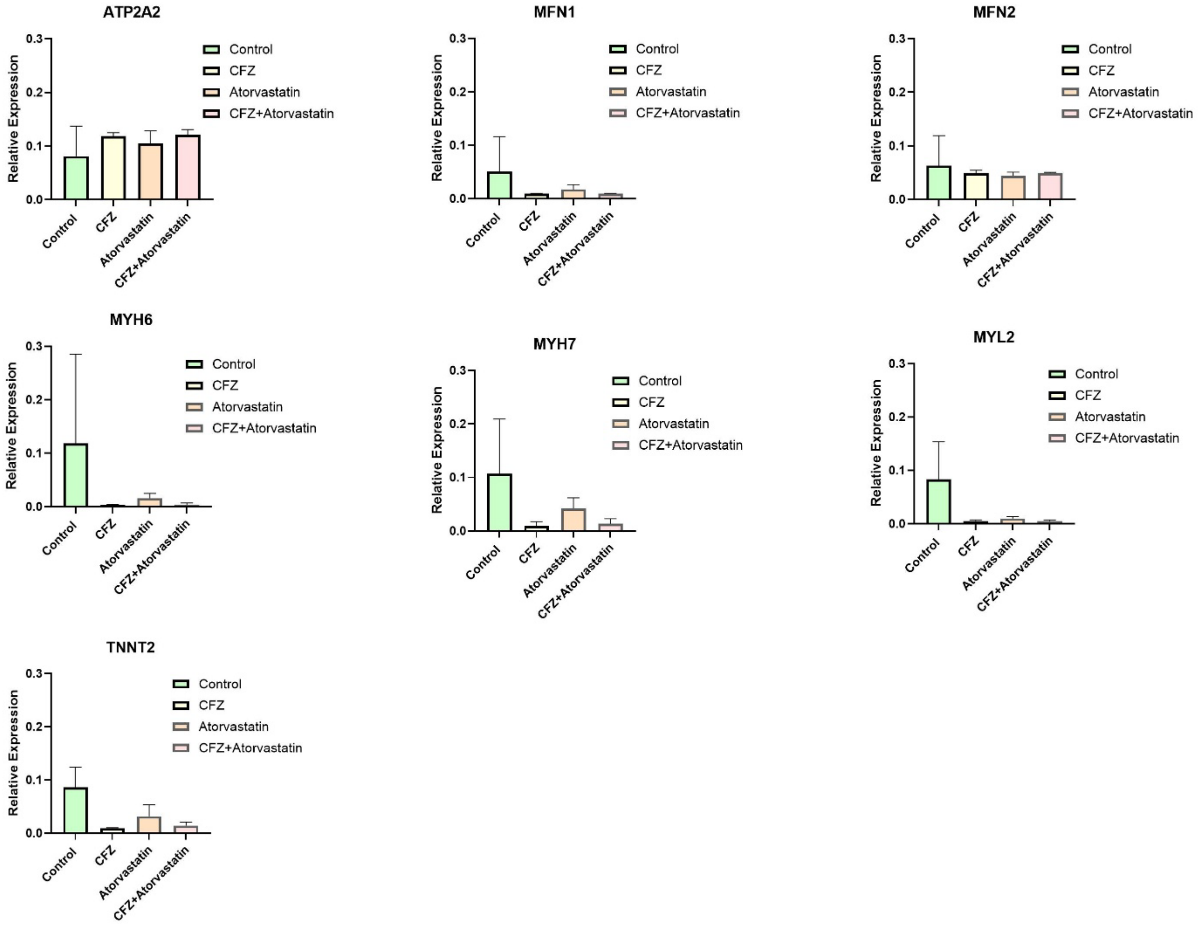

Supplement: Supplementary file 1 [file ijms-27-01358-s001.zip › ijms-4085913-supplementary figures.pdf]
